# Combined PLR and VAS for patient self-perception in rheumatoid arthritis: a retrospective study

**DOI:** 10.3389/fmed.2026.1836171

**Published:** 2026-07-01

**Authors:** Shengfeng Liu, Jian Liu, Xueni Cheng, Yang Li

**Affiliations:** 1 The First Affiliated Hospital of Anhui University of Chinese Medicine, Hefei, China; 2 Anhui Provincial Key Laboratory for Applied Basic and Clinical Translational Research on Rheumatologic Diseases in Traditional Chinese Medicine, Hefei, Anhui, China

**Keywords:** PLR, retrospective study, rheumatoid arthritis, SPP, VAS

## Abstract

**Objective:**

To explore the independent and synergistic predictive value of platelet/lymphocyte ratio (PLR) and visual analogue scale (VAS) for multidimensional self-perception of patient (SPP) in rheumatoid arthritis (RA), and to support high-risk population stratification.

**Methods:**

A total of 708 RA patients were enrolled and categorized into four groups according to the median levels of PLR and VAS. Correlation and multivariate logistic regression analyses were adopted to evaluate their associations with SPP.

**Results:**

PLR and VAS were closely correlated with multidimensional SPP abnormalities. High VAS served as an independent risk factor for SPP deterioration. Combined high PLR and high VAS exhibited a prominent synergistic effect, greatly increasing the risk of impaired mental health and TCM spleen deficiency with dampness syndrome.

**Conclusion:**

Combined PLR and VAS assessment offers synergistic predictive value for SPP deterioration in RA. The dual-high phenotype can serve as a simple auxiliary marker for identifying high-risk RA patients to facilitate targeted monitoring and personalized management, with further prospective validation still required.

## 1 Introduction

Rheumatoid arthritis (RA) is a chronic and systemic autoimmune disease characterized by persistent synovial inflammation, which leads to gradual joint destruction, functional impairment, and severe disability (1). Approximately 0.5 to 1% of the global population suffers from this disease, and the prevalence rate among women is 2 to 3 times higher (2). RA has imposed a significant social and economic burden due to the decline in patients’ working ability and the increase in medical resources.

In addition to the existing biomarkers, new hematological indicators that reflect systemic inflammation and immune status have attracted much attention due to their prognostic value in RA. The platelet-to-lymphocyte ratio (PLR) calculated from routine complete blood counts integrates the effects of platelets and lymphocytes (3). An increase in PLR indicates an imbalance between pro-inflammatory and pro-thrombotic states: activated platelets release cytokines such as IL-1*β* and TGF-β, which drive the formation of synovial vascular (4). Lymphocyte depletion weakens anti-inflammatory regulation (5). PLR, as a quantifiable and low-cost indicator derived, provides a supplementary perspective to traditional inflammatory markers, and is particularly helpful in identifying the subgroups of RA with latent inflammatory activity (6). The overall disease activity or pain visual analogue scale (VAS) evaluated by the patients provides a direct and quantifiable measure of the patients’ subjective experience of the burden of the disease (7). VAS is the cornerstone of the composite disease activity index, and is recommended by EULAR as a key patient-reported outcome (PRO) for monitoring treatment response and determining treatment goals (8). Self-Perception of Patient (SPP) is a comprehensive subjective assessment system based on an individual’s overall evaluation of their physical, psychological and social functional status. Its core lies in quantifying the difference between an individual’s health expectations and actual experiences. The greater the difference, the more significant the negative perception of the patient and the lower the quality of life (9). SPP is a multi-dimensional evaluation framework that has been applied in RA cohorts rather than a single composite scale.

This study employed the standard tools recommended by the International Consortium for Health Outcomes Measurement (ICHOM) to assess SPP, including the Short Form 36 Health Survey (SF-36) (10). Self-rating Anxiety Scale (SAS) (11) and the Self-Rating Depression Scale (SDS) (12). To highlight the characteristics of traditional Chinese medicine and to dynamically track the impact of syndromes, we have specially integrated the traditional Chinese medicine syndrome scoring system, and systematically evaluatedSyndrome of Dampness Heat (SDH) (13), Syndrome of Blood Stasis (SBS) (14) and Syndrome of Dampness Stage due to spleen deficiency (SDSSD) (15). The inclusion of these TCM syndromes is grounded in a pathophysiological rationale: elevated PLR reflects a pro-inflammatory and pro-thrombotic state, which closely parallels the TCM pathogenesis of “Dampness-Heat” (inflammatory exudation and swelling) and “Blood Stasis” (microcirculatory disturbance and hypercoagulability). Furthermore, chronic systemic inflammation can impair digestive and metabolic functions, leading to fatigue, poor appetite, and heavy limbs—symptoms that align with the TCM concept of “Spleen Deficiency with Dampness Accumulation.” Thus, PLR and VAS may serve as objective surrogates linking Western inflammatory markers to TCM syndrome patterns. The combined action of these tools can comprehensively reflect the actual living conditions of patients, rather than just what can be reflected by the number of joints and laboratory test results.

Although PLR and VAS are, respectively, associated with the severity and prognosis of RA, the potential interactions between them and the comprehensive influence on SPP have not been fully elucidated. This retrospective study aims to fill these key gaps in the following ways: (1) to study the individual and combined associations between PLR and VAS and SPP indicators; (2) to explore the potential mediating or regulatory roles between PLR, VAS, and SPP results; and (3) to evaluate the predictive value of PLR and VAS alone or in combination for SPP deterioration. By clarifying these complex interactions, this study may identify high-risk patients based on the PLR/VAS indicators, providing a basis for developing more comprehensive and personalized management strategies that combine biomedical and traditional Chinese medicine perspectives.

## 2 Materials and methods

### 2.1 Data sources

Clinical data of patients diagnosed with rheumatoid arthritis (RA) were obtained from the electronic medical record system of the Department of Rheumatology at the First Affiliated Hospital of Anhui University of Chinese Medicine in China. The diagnostic criteria for RA were based on the latest classification standards and scoring systems proposed by the American College of Rheumatology (ACR) and the European League Against Rheumatism (EULAR) in 2010 (16). When at least one joint is swollen, painful and accompanied by synovitis, it can be diagnosed as RA; exclude arthritis caused by other diseases, and have typical imaging manifestations of bone destruction. In addition, RA can also be diagnosed by scoring the four components: joint involvement, serological indicators, duration of synovitis, and acute time response markers, with a total score of 6 or above. The indicators included in this study are general indicators, laboratory indicators, main research indicators, and SPP indicators. General indicators include: gender, age, BMI, disease duration, and laboratory indicators include: PLT, LYM, MPV, PCT, PDW, RF, IgA, IgG, IgM, Hs-CRP, CCP, C3, C4, IL-6.

This study has been registered on the Chinese Traditional Medicine Clinical Trial Registration Platform (ITMCTR) (registration number: ITMCTR2025001241). The study adhered to the provisions of the Helsinki Declaration and was approved by the Ethics Committee of the First Affiliated Hospital of Anhui University of Chinese Medicine (ethics number: 2025AH-57).

### 2.2 Inclusion and exclusion criteria

*Inclusion criteria*: (1) Patients aged ≥18 years; (2) Fulfilled the 2010 ACR/EULAR classification criteria for RA; (3) Had complete medical records including at least one complete set of SPP assessments (SF-36, SAS, SDS, and TCM syndrome scores) and laboratory tests (complete blood count for PLR, VAS score) within the study period; (4) No change in disease-modifying anti-rheumatic drug (DMARD) regimen within 4 weeks prior to data collection.

*Exclusion criteria*: (1) Patients with other concurrent autoimmune diseases (e.g., systemic lupus erythematosus, psoriatic arthritis); (2) Active infection or malignancy; (3) Severe cardiovascular, hepatic, or renal dysfunction; (4) Pregnancy or lactation; (5) Use of glucocorticoids >10 mg/day prednisone equivalent within the past 4 weeks; (6) Missing data for key study variables (PLR, VAS, or any SPP component) after attempting data retrieval.

### 2.3 SPP indicators

SPP is a multi-dimensional health concept integrating physiological, psychological and traditional Chinese medical syndromes. This study comprehensively evaluated SPP using the following standardized tools: Health-related Quality of Life (SF-36): covering eight dimensions including physical function (PF), role-physical (RP), bodily pain (BP), general health (GH), vitality (VT), social function (SF), emotional function (RE), and mental health (MH); psychological state: using the Self-Rating Anxiety Scale (SAS) and the Self-Rating Depression Scale (SDS); Traditional Chinese Medicine syndrome scoring: including damp-heat syndrome (SDH), blood stasis syndrome (SBS), and spleen deficiency with dampness syndrome (SDSSD). These syndromes reflect specific syndrome clusters under the traditional Chinese medical theory and make a unique contribution to the assessment of SPP in integrated medical practice.

For the VAS (Visual Analogue Scale) measurement, patients were asked: “Please rate your average level of pain and overall disease activity over the past week” using a 0–10 numerical rating scale (equivalent to a 0–10 cm visual analogue scale), where 0 represented “no pain/no disease activity” and 10 represented “worst imaginable pain/most severe disease activity.” Thus, VAS in this study reflects a composite of pain intensity and patient global assessment of disease activity. This VAS score was recorded during each clinical visit and was collected independently of the physician’s joint examination.

For TCM syndrome quantification, each of the three syndromes (SDH, SBS, SDSSD) was assessed using validated, syndrome-specific scoring instruments. These instruments consist of a checklist of typical signs and symptoms (e.g., for SDSSD: fatigue, poor appetite, loose stools, heavy limbs, a greasy tongue coating, and a soggy pulse). Each item is scored on a 0–3 point scale (0 = absence, 1 = mild, 2 = moderate, 3 = severe). The total score for each syndrome was calculated as the sum of item scores. All TCM syndrome scores were assigned independently by two attending-level or senior TCM physicians who were blinded to patients’ PLR and VAS levels. In case of disagreement, a third senior TCM physician was consulted to reach consensus. These syndromes reflect specific syndrome clusters under the traditional Chinese medical theory and make a unique contribution to the assessment of SPP in integrated medical practice.

### 2.4 Indicator calculation and definition

The calculation formula for PLR is: PLR = PLT/LYM. The calculation formula for BMI is: BMI = weight (kg) ÷ height (m)^2^ [The VAS score was recorded as described in section 2.2, using a 0–10 scale]. We will divide the PLR and VAS levels by the median (VAS median = 5.7). Those above the median are classified as high levels, and those below the median are classified as low levels. For TCM syndromes, the median score of each syndrome (derived from the sum of item scores) was used as the cutoff to define “high” (above median) and “low” (below median) groups. The defined ranges for each clinical indicator refer to the laboratory standards of the First Affiliated Hospital of Anhui University of Traditional Chinese Medicine. The normal level of SF-36 is 50 or above, the normal level of SAS and SDS is 50 or above (17). And the TCM syndrome score is divided according to the median.

### 2.5 Correlation analysis

A non-parametric rank correlation measurement method–Spearman correlation–was adopted to study the statistical dependence relationship between the rankings of two variables. The Spearman correlation coefficient between two variables is equivalent to the Pearson correlation coefficient between the rank values of these variables, and is used to evaluate monotonic relationships (whether linear or non-linear). In this study, Spearman correlation analysis was used to explore the correlations between PLR, VAS, clinical indicators and SPP indicators.

### 2.6 Restricted cubic spline plot

The Restricted Cubic Spline Plot (RCS) is a type of chart used to display the Nonlinear relationship between the independent variable and the dependent variable. It is mainly used to fit the Nonlinear relationship between the independent variable and the dependent variable. By fitting multiple Linear functions within different intervals of the independent variable, it captures the changing trends in the data. Each segment is connected by nodes, and the position and number of nodes can be adjusted according to the data distribution to improve the accuracy of the fitting. To flexibly model the potential Nonlinear relationship between continuous variables such as PLR, VAS, and SPP indicators, we adopted the Restricted Cubic Spline. Four nodes were used to generate the spline basis functions. After model fitting, the results were explained by drawing relationship curves, conducting Nonlinear likelihood ratio tests, and analyzing the curve shapes.

### 2.7 Logistic regression analysis

A logistic regression model was used to predict the relevant protective and risk factors of the SPP index. Multivariable models were adjusted for the following covariates: age, sex, body mass index (BMI), disease duration, rheumatoid factor (RF), anti-cyclic citrullinated peptide antibody (CCP), high-sensitivity C-reactive protein (Hs-CRP), interleukin-6 (IL-6), complement 3 (C3), complement 4 (C4), and serum immunoglobulin levels (IgA, IgG, IgM). These covariates were selected *a priori* based on clinical relevance and known associations with RA disease activity, inflammatory status, or SPP outcomes in the literature (1, 3, 5, 12). Specifically, age and sex are demographic factors that influence disease presentation and perception; BMI and disease duration reflect long-term disease burden; RF, CCP, Hs-CRP, and IL-6 are established markers of RA inflammatory activity; and complement and immunoglobulin levels capture immunological aspects that may confound the relationships between PLR, VAS, and SPP. Notably, medication use. In the binary logistic regression analysis, a *p*-value less than 0.05 indicated a statistically significant relationship. An odds ratio (OR) greater than 1 indicated a risk factor, while an OR less than 1 indicated a protective factor. The specific formula is as follows (18):


$$\log\mathrm{itP}=\ln[\frac{P}{1-P}]=a+b_{1}x_{1}+b_{2}x_{2}+\cdots +b_{n}x_{n}$$

$$\text{OR}=\frac{\left[P_{1}/(1-P_{1})\right]}{P_{0}/(1-P_{0})}$$

### 2.8 Mediation analysis

**Figure 1:**
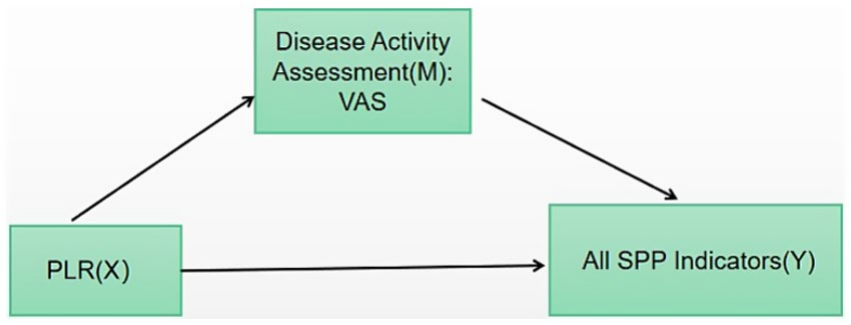
Mediation analysis effect plots.

Mediation analysis (MA) is a statistical method used to study how an independent variable (X) affects the dependent variable (Y) through one or more intermediate variables (referred to as mediating variables, M). It helps researchers understand the potential mechanism or process by which the independent variable influences the dependent variable. Notably, owing to the retrospective observational design of our study, all mediation analyses here are exploratory and hypothesis-generating and cannot confirm causal pathways. To test whether VAS plays a role in the PLR-mediated SPP results, a causal mediation analysis was conducted according to the method proposed by VanderWeele (19) (Figure 1).

### 2.9 Association rule analysis

The association rule analysis was conducted using the Apriori algorithm in IBM SPSS Modeler 18.0 software. The minimum support for the association rules was set at 45%, the minimum confidence at 50%, and the lift value was greater than 1. In the association rule analysis of PLR, VAS, and SPP indicators, high PLR, high VAS, and abnormal SPP indicators were represented by T, otherwise by F. In the association rule analysis combining PLR and VAS, or with SPP indicators, when both PLR and VAS were high and the SPP indicator was abnormal, it was represented by T, otherwise by F. The formula of the Apriori algorithm in the association rules is as follows (20):


$$\text{support}(x\to Y)=\sigma \frac{\left(X\cup Y\right)}{N}$$

$$\text{confidence}(X\to Y)=\sigma \frac{\left(X\cup Y\right)}{\sigma (X)}$$

$$\text{lift}(X\to Y)=\text{confidence}\frac{\left(X\to Y\right)}{\sigma (Y)}$$

### 2.10 Statistical analysis

Statistical analysis was conducted using IBM SPSS software. For data description, for normally distributed data, the mean ± standard deviation method was used, and for non-normally distributed data, the quartile method was employed. For any data group that did not conform to a normal distribution, the Kruskal-Wallis non-parametric test was adopted. For the comparison of count data, the chi-square test was used. Image drawing was performed using GraphPad Prism 8.0.2 software. A *p*-value < 0.05 was considered statistically significant.

## 3 Result

### 3.1 The baseline characteristics of the participants

A total of 708 participants (82% female) were enrolled, with a median age of 59.00 (53.00, 69.75). Participants were divided into four subgroups according to the median of PLR and VAS: high PLR/high VAS, high PLR/low VAS, low PLR/high VAS, low PLR/low VAS. Baseline demographic, clinical, SPP and laboratory parameters are summarized in Tables 1, 2. Significant intergroup differences were observed in disease duration, SF-36 dimensions, SAS, SDS, TCM syndrome scores (SDH, SDSSD, SBS), as well as RF, Hs-CRP and IL-6 (all *p* < 0.05). Other routine blood and immunological indicators showed no significant differences.

**Table 1:** Baseline characteristics of participants.

Basic Characteristics	Total	High PLR and High VAS	High PLR and Low VAS	Low PLR and High VAS	Low PLR and Low VAS	*p* value
*n*	708	206	147	164	191	
Sex						0.54
Woman	580	171	120	130	159	
Man	128	35	27	34	32	
Age	59.00 (53.00,69.75)	60.00 (54.00,70.00)	58.00 (51.00,68.00)	60.00 (54.00,71.00)	58.00 (51.00,69.00)	0.33
BMI	22.27 (20.00,24.22)	22.26 (20.19,24.44)	21.95 (19.89,23.95)	22.10 (19.60,24.11)	22.48 (20.57,25.08)	0.31
Course of the disease	10.00 (3.00,19.00)	10.00 (3.00,20.00)	8.00 (3.00,18.25)	10.00 (5.00,20.00)	7.00 (3.00,10.00)	<0.05

**Table 2:** The SPP and laboratory characteristics of the participants.

Indicators	Total	High PLR and High VAS	High PLR and Low VAS	Low PLR and High VAS	Low PLR and Low VAS	*P* value
PF	30.00 (25.00,40.00)	30.00 (20.00,35.00)	35.00 (25.00,50.00)	30.00 (25.00,35.00)	35.00 (30.00,50.00)	<0.05
RP	25.00 (0.00,25.00)	25.00 (0.00,25.00)	0.00 (0.00,25.00)	25.00 (0.00,25.00)	0.00 (0.00,25.00)	<0.05
BP	31.00 (30.99,41.00)	30.99 (22.00,41.00)	31.00 (30.99,41.00)	31.00 (22.00,41.00)	41.00 (30.99,41.00)	<0.05
GH	30.00 (20.00.35.00)	25.00 (20.00.31.00)	30.00 (25.00.35.00)	30.00 (20.00.35.00)	30.00 (25.00.35.00)	<0.05
VT	40.00 (35.00,45.00)	35.00 (25.00,41.25)	40.00 (35.00,45.00)	40.00 (30.00,45.00)	45.00 (40.00,50.00)	<0.05
SF	50.00 (37.50,62.50)	50.00 (25.00,50.00)	50.00 (37.50,62.50)	50.00 (37.50,50.00)	50.00 (50.00,62.50)	<0.05
RE	33.33 (0.00,33.33)	33.33 (33.33,33.33)	33.33 (0.00,66.66)	33.33 (33.33,33.33)	33.33 (0.00,66.66)	<0.05
MH	40.00 (36.00,48.00)	36.00 (32.00,44.00)	44.00 (40.00,52.00)	36.00 (36.00,44.00)	48.00 (40.00,56.00)	<0.05
SAS	53.75 (51.25,56.25)	55.00 (52.50,60.00)	52.50 (50.00,55.00)	55.00 (52.50,58.44)	52.50 (50.00,55.00)	<0.05
SDS	60.00 (57.50,65.00)	61.25 (57.50,67.50)	60.00 (56.25,63.75)	60.63 (57.50,65.00)	60.00 (56.25,63.75)	<0.05
SDH	14.00 (12.00,16.00)	15.00 (13.00,18.00)	13.00 (11.00,15.00)	15.00 (13.00,18.00)	13.00 (11.00,14.00)	<0.05
SDSSD	12.00 (9.00,14.00)	14.00 (11.00,16.25)	10.00 (8.00,13.00)	12.00 (10.00,16.00)	10.00 (8.00,12.00)	<0.05
SBS	7.00 (5.00,8.00)	6.00 (7.00,9.00)	6.00 (4.00,7.00)	7.00 (6.00,8.00)	6.00 (4.00,7.00)	<0.05
PCT	0.0025 (0.0021,0.0031)	0.0027 (0.0023,0.0033)	0.0027 (0.0023,0.0032)	0.0023 (0.0019,0.0028)	0.0027 (0.0023,0.0033)	0.63
MPV	10.50 (9.80,11.30)	10.10 (9.50,10.90)	10.30 (9.80,10.90)	10.80 (9.93,11.80)	10.70 (10.20,11.50)	0.47
PDW	11.80 (10.50,13.58)	11.10 (9.50,10.90)	11.40 (10.40,12.70)	12.60 (10.83,14.9)	12.50 (11.20,14.80)	0.11
RF	106.50 (37.48,253.88)	124.99 (48.18,373.28)	94.30 (39.20,208.20)	100.60 (28.68,241.18)	112.00 (35.70,227.5)	<0.05
IgA	2.85 (2.08,4.00)	2.93 (2.08,4.03)	2.89 (2.14,4.08)	2.81 (2.17,3.86)	2.74 (2.02,4.06)	0.81
IgG	12.34 (10.12,15.09)	12.21 (10.07,15.08)	12.69 (10.53,15.72)	12.03 (9.62,14.74)	12.40 (10.29,15.12)	0.38
IgM	1.20 (0.87,1.74)	1.24 (0.88,1.92)	1.25 (0.94,1.79)	1.08 (0.79,1.65)	1.21 (0.91,1.59)	0.23
Hs-CRP	13.86 (3.55,36.61)	24.74 (7.35,55.43)	17.05 (5.85,44.56)	9.01 (2.01,27.13)	6.40 (2.13,25.65)	<0.05
CCP	95.30 (15.40,300.50)	101.50 (17.73,281.75)	91.20 (19.10,302.65)	106.50 (10.68,320.75)	82.66 (14.00,295.00)	0.88
C3	1.27 (1.12,1.45)	1.28 (1.14,1.46)	1.31 (1.18,1.49)	1.24 (1.11,1.46)	1.23 (1.10,1.41)	0.63
C4	0.31 (0.24,0.39)	0.31 (0.25,0.39)	0.33 (0.25,0.41)	0.31 (0.24,0.39)	0.30 (0.24,0.38)	0.46
IL-6	18.62 (5.54,53.25)	22.02 (9.20,75.10)	21.69 (5.54,58.22)	17.74 (4.26,52.62)	13.19 (4.52,35.86)	<0.05

### 3.2 Descriptive statistics of SPP indicators in the overall cohort

SF-36 floor and ceiling effects are presented in Supplementary Table 2. Obvious floor effects were found in RP and RE, indicating a high proportion of patients suffered from physical and emotional role limitation, while no prominent ceiling effects were observed for most dimensions. As shown in Supplementary Table 3, the overall prevalence of anxiety and depression was 78.4 and 96.2%, respectively, reflecting a severe psychological burden in this RA cohort. Supplementary Table 4 showed moderate-to-severe TCM syndrome burden in the overall population, with median scores of SDH, SDSSD and SBS at 14, 12, and 7, respectively.

### 3.3 Correlation analysis between clinical indicators and SPP indicators

**Figure 2:**
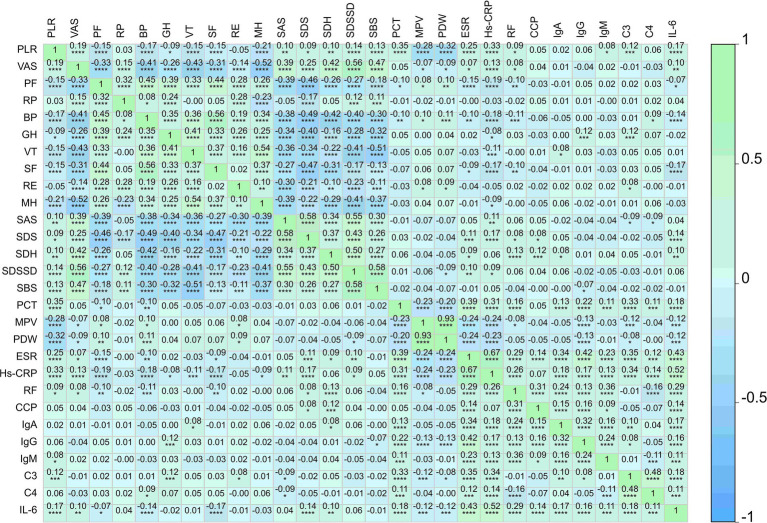
Heatmap for correlation analysis of PLR, VAS, clinical indicators and SPP indicators.

Spearman correlation analysis showed that PLR was positively correlated with VAS, negatively correlated with most SF-36 dimensions and MH, and positively correlated with SAS, SDS and three TCM syndromes. Among them, the negative correlation between PLR and MH was the most prominent. Compared with PLR, VAS exhibited much broader and stronger correlations with SPP indicators. VAS was strongly negatively correlated with MH and multiple quality-of-life dimensions, and strongly positively correlated with SAS, SDS, especially SDSSD. Inflammatory markers Hs-CRP and IL-6 were also significantly correlated with VAS and TCM syndrome indicators (Figure 2; Supplementary Figure 1).

### 3.4 The linear/nonlinear correlation between PLR or VAS and SPP indicators

**Figure 3:**
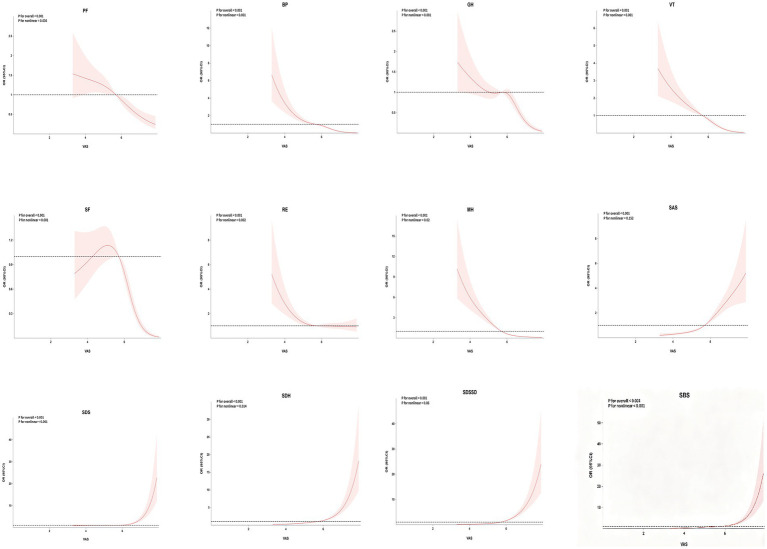
RCS analysis of PLR and SPP indicators.

**Figure 4:**
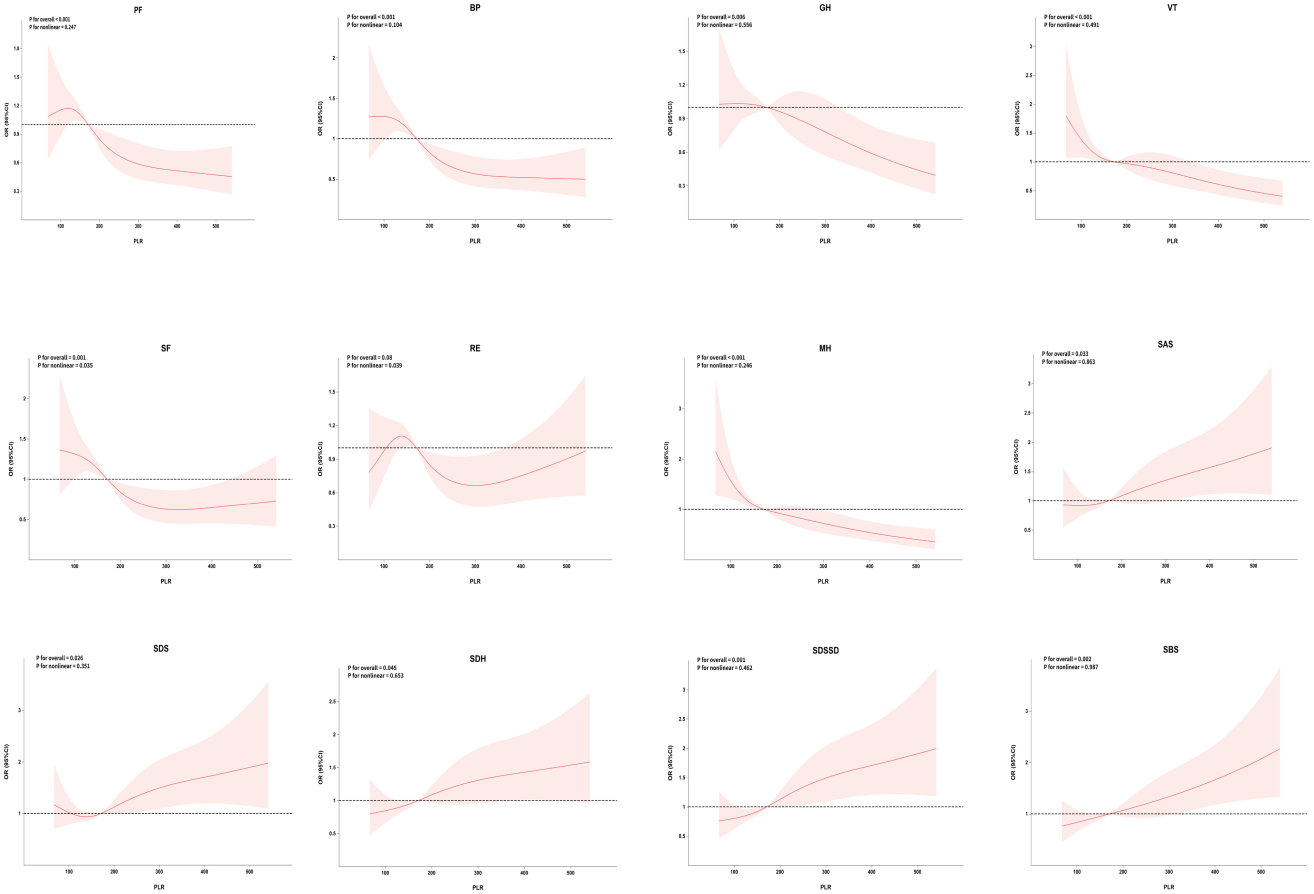
RCS analysis of VAS and SPP indicators.

Restricted cubic spline (RCS) analysis revealed distinct dose–response patterns between PLR/VAS and SPP indicators. PLR mainly showed linear trends with most SPP outcomes, while only SF presented a nonlinear relationship. In contrast, VAS was dominated by nonlinear dose–response curves for most indicators, except SAS and SDSSD, which followed linear patterns (Figures 3, 4). All detailed *p*-values and linear/nonlinear test results are listed in Supplementary Table 1.

### 3.5 Independent risk effects of PLR and VAS

**Figure 5:**
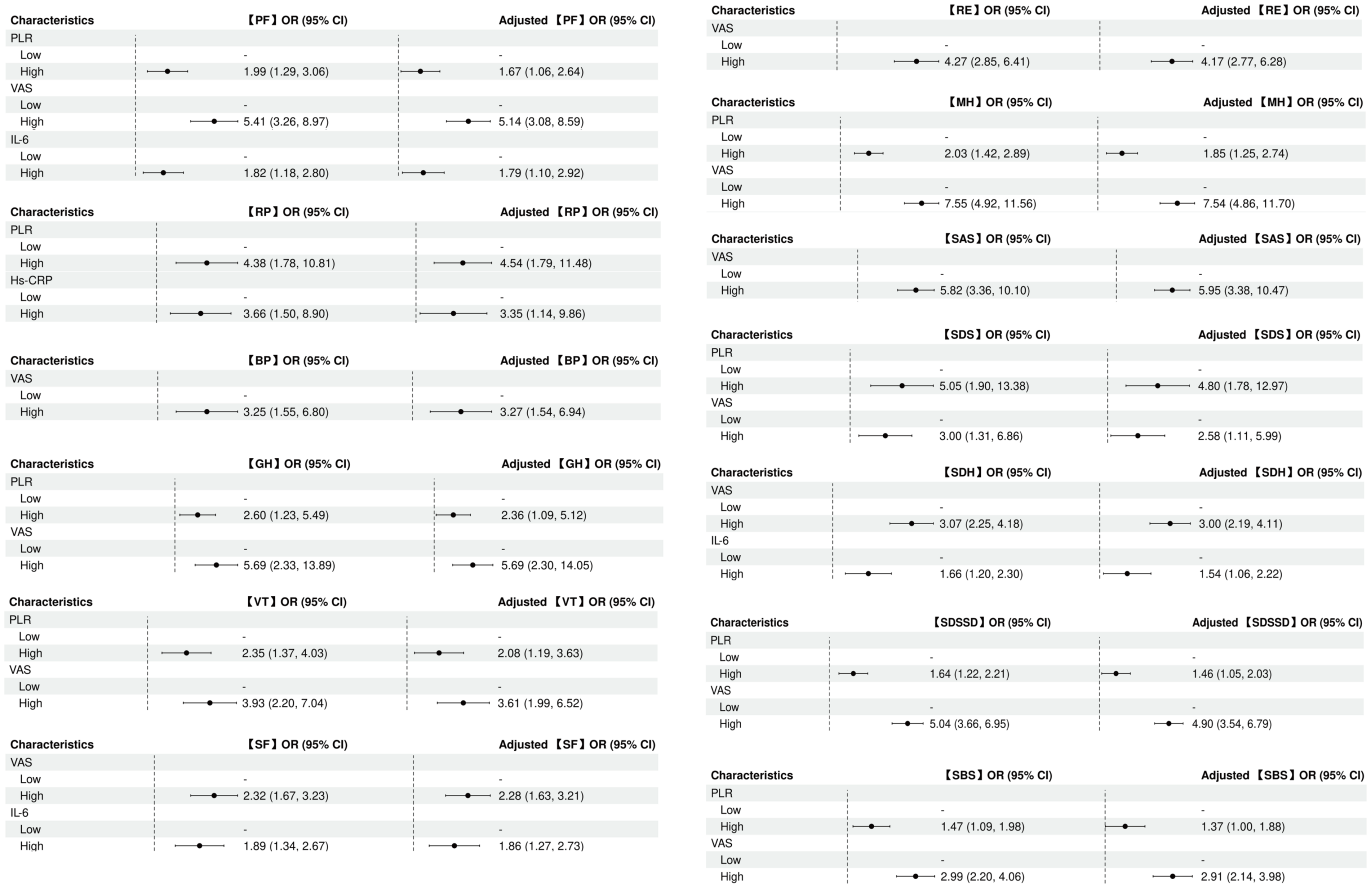
Logistic regression analysis of the SPP indicators using separate PLR and separate VAS separately.

Multivariate logistic regression analysis demonstrated that high VAS served as the strongest independent risk factor for nearly all SPP abnormalities, with adjusted OR ranging from 2.28 to 7.54. High PLR also independently increased the risk of impaired physical function, role limitation and mental health deterioration. In addition, elevated IL-6 and Hs-CRP were independent risk factors for partial SPP dimensions (Figure 5; Supplementary Table 5).

### 3.6 Synergistic predictive effect of combined PLR and VAS

**Figure 6:**
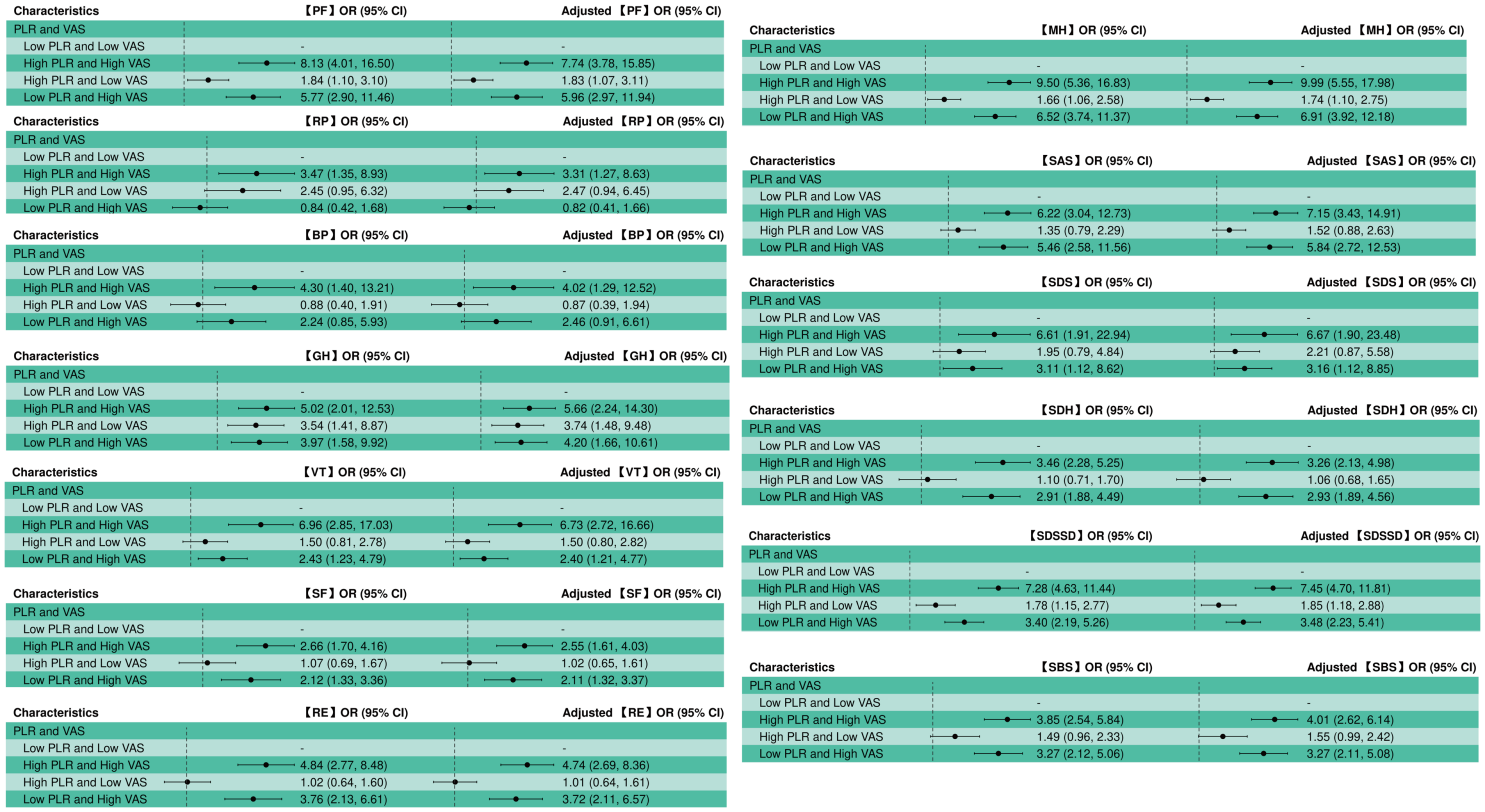
Logistic regression analysis of the combined PLR and VAS indicators versus the SPP indicator.

Compared with the low PLR + low VAS reference group, the high PLR + high VAS group showed significantly elevated risks across most SPP dimensions, with obvious synergistic effects. The strongest synergistic risk was observed for poor mental health (MH: OR = 9.99) and spleen deficiency with dampness syndrome (SDSSD: OR = 7.45), which were far higher than the risks in the single-high subgroups (Figure 6). This indicated that the co-elevation of inflammatory biomarker PLR and subjective pain VAS substantially amplified the risk of SPP deterioration. Detailed combined risk values are shown in Supplementary Table 6.

### 3.7 Analysis of association rules between PLR or VAS and combined indicators and SPP indicators

Association rule analysis further confirmed that VAS had better predictive performance for MH than PLR alone. Importantly, the combination of PLR and VAS achieved over 90% prediction confidence for TCM syndromes, with lift values between 1.18–1.26, showing excellent predictive stability (Supplementary Figure 2). All support, confidence and lift parameters are detailed in Supplementary Tables 7, 8.

### 3.8 Analysis of the mediating path through which PLR affects the SPP indicators via VAS

To explore the potential mediating role of VAS between PLR and various SPP indicators, we conducted a series of mediation effect analyses. All detected mediating associations only reflect statistical correlations rather than proven causal relationships; these results are hypothesis-generating findings instead of definitive mechanistic evidence. As shown in Supplementary Table 9, VAS played a significant partial mediating role in the relationships between PLR and PF, overall GH, VT, SF, SAS, SDS, and SDH. VAS also played a significant complete mediating role in the relationship between PLR and MH and SDSSD. In the relationships between PLR and RP and RE, the analysis showed that the mediating effect of VAS did not hold.

### 3.9 Subgroup analysis

**Figure 7:**
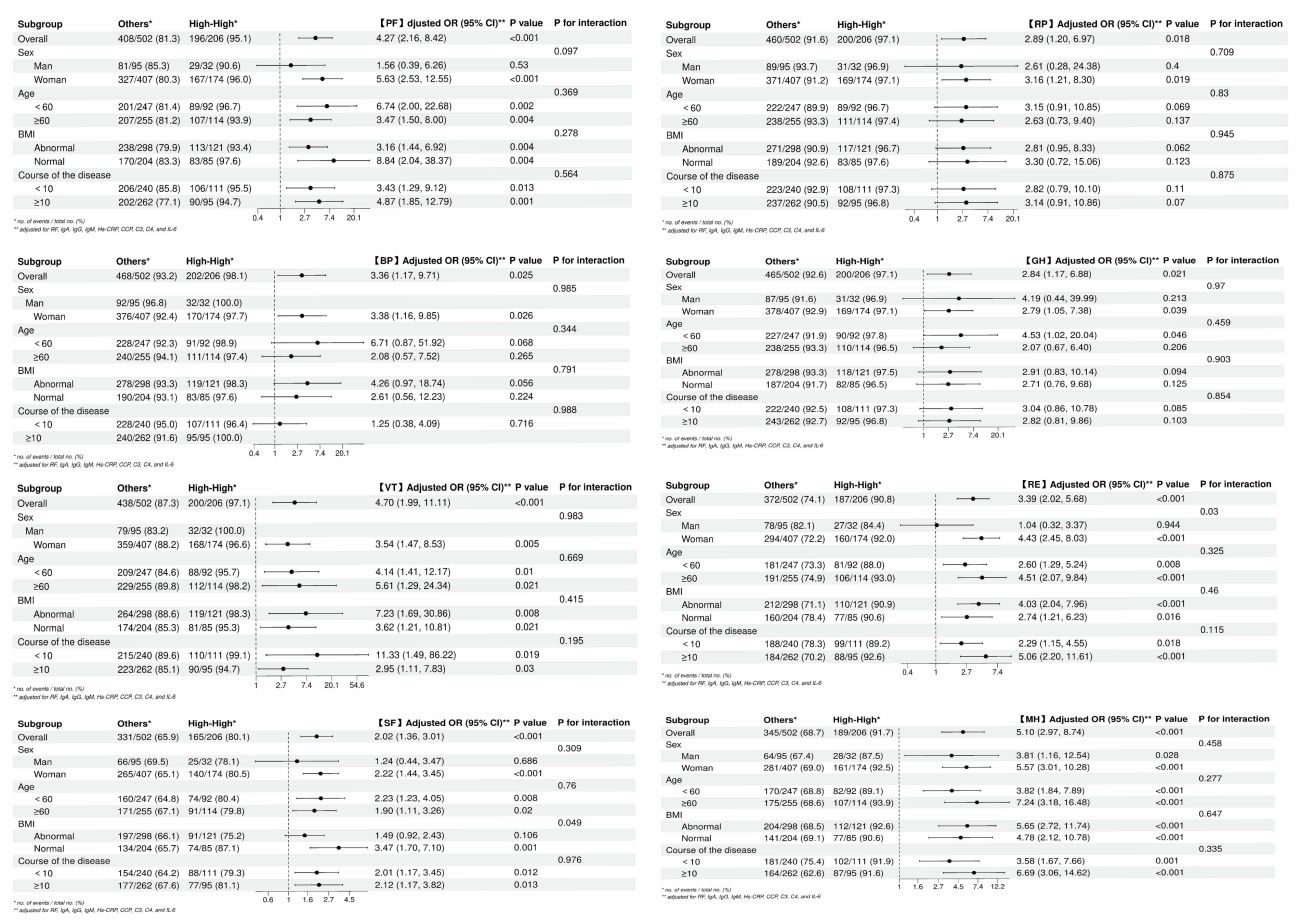
Subgroup analysis and interaction forest plot.

**Figure 8:**
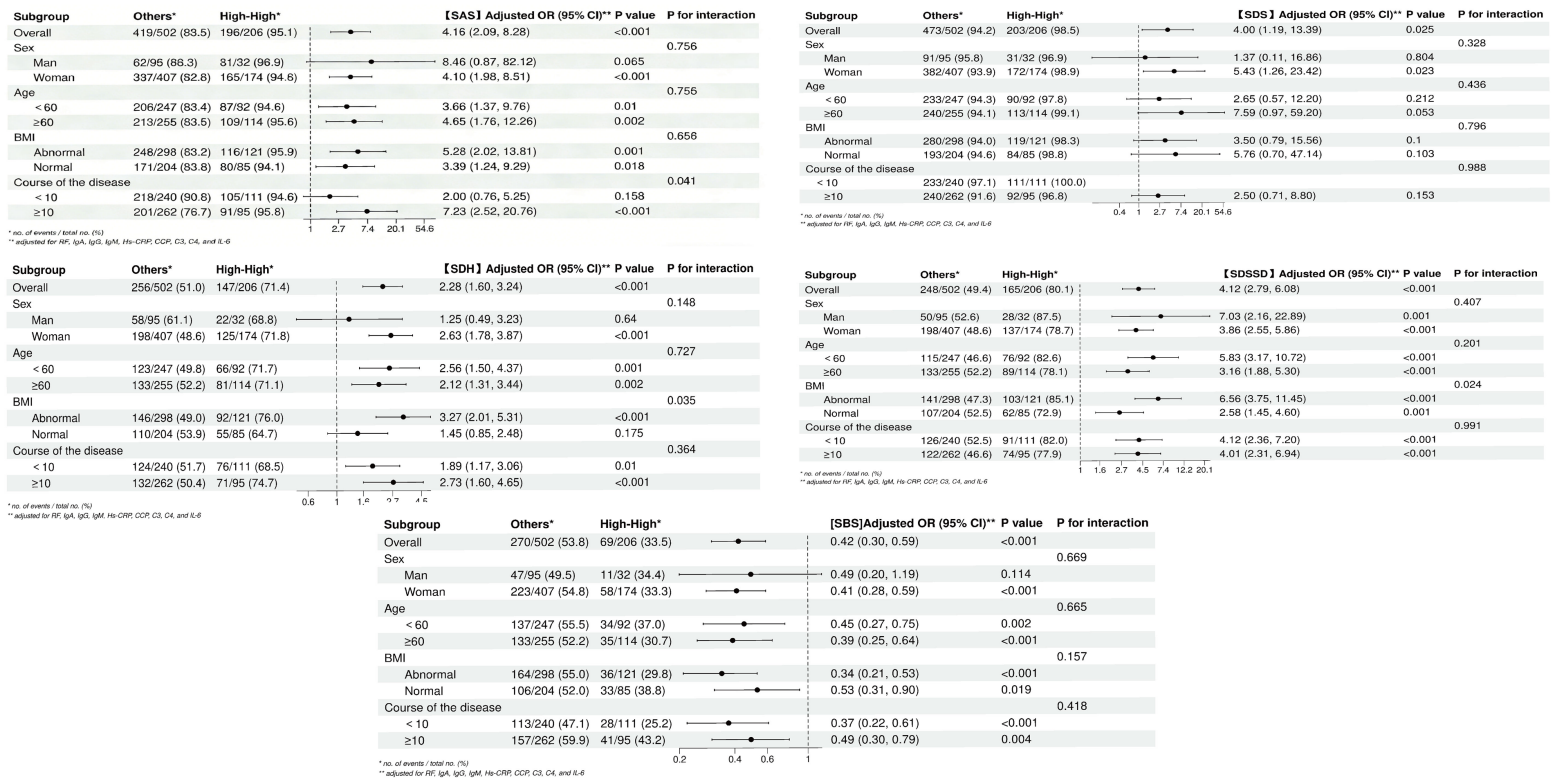
Subgroup analysis and interaction forest plot.

Correlation analysis showed that gender, age, disease duration, and BMI were closely correlated with PLR, VAS and SPP indicators (Supplementary Figure 3). Therefore, we performed subgroup and interaction analyses to explore the differences in the associations between high PLR + high VAS and SPP outcomes across different populations. In the overall population, the high-high group exhibited significantly elevated risks for most SPP dimensions (all *p* < 0.05), with consistent and stable associations in both age subgroups (<60 years and ≥60 years). Although the gender interaction was only significant for RE (*p* = 0.03), the increased risks of most SPP abnormalities were mainly observed in females, while the associations were weaker or non-significant in males. Disease duration exerted a notable modifying effect. A significant interaction was found for SAS (*p* = 0.041), and patients with a disease duration≥10 years had a much higher risk than those with shorter disease courses. Similarly, higher risk trends were also observed in RE, MH and SBS among long-duration patients. Significant BMI interactions were detected for SF, SDH and SDSSD (*p* < 0.05). The association was stronger in normal BMI individuals for SF and SDH, whereas the risk of SDSSD was more prominent in the abnormal BMI group. Overall, the combined high PLR and high VAS conferred higher susceptibility to SPP deterioration, particularly in females, patients with long disease duration (≥10 years), and those with abnormal BMI (Figures 7, 8).

## 4 Discussion

RA is an autoimmune inflammatory condition that primarily affects the joints and periarticular soft tissue (21). However, in reality, the impact of RA extends far beyond the joint area: the systemic inflammatory response can activate endothelial cells, platelets and the neuroendocrine system, leading to multi-organ damage and systemic symptoms (22). This systemic pathological process is directly reflected in the core indicators of this study: the elevated PLR indicates an imbalance between inflammation and thrombosis (23). The VAS score not only reflects the intensity of local inflammation in the joint (24). It is also closely related to the pain hypersensitivity caused by central sensitization (25). The deterioration of SPP represents the ultimate manifestation of the aforementioned pathological process, encompassing SF-36, SAS/SDS, and traditional Chinese medicine syndromes.

PLR, as a systemic inflammatory marker, plays a central role in the deterioration of SPP. This study confirmed that PLR is significantly correlated with the multi-dimensional damage of SPP in RA patients. An elevated PLR indicates excessive activation of platelets, and platelet activation has a direct damaging effect (26). Activated platelets release pro-inflammatory factors and angiogenic factors, directly promoting the formation of synovial vascularnevus, resulting in joint structure damage (27). This can explain the strong correlation between PLR and the decline in PF. At the same time, pro-inflammatory factors can increase the permeability of the blood–brain barrier (28). And activate the afferent fibers of the vagus nerve or promote the transmission of inflammatory signals from peripheral immune cells to the brain (29). This systemic inflammatory state eventually leads to central nervous inflammation and dysfunction of the neurotransmitter system (30). This is highly consistent with the positive correlation between PLR and SAS in this study, as well as the linear negative correlation between MH. On the other hand, lymphocyte exhaustion leads to an imbalance in immune regulation. The reduction of lymphocytes will result in a decrease in the secretion of anti-inflammatory factor IL-10, which is unable to effectively inhibit pro-inflammatory factors such as TNF-*α* (31). This imbalance not only aggravates joint inflammation, but also affects the limbic system through the “brain-immune axis”: animal experiments have shown that TNF-α can inhibit hippocampal neurogenesis and impair emotional regulation functions (32). The mechanism supports the independent predictive value of PLR for MH. The pro-thrombotic state is associated with the traditional Chinese medicine “blood stasis syndrome.” This study found that PLR was linearly positively correlated with the SBS. From the perspective of modern medicine, an increase in platelets indicates a possible hypercoagulable state, leading to microcirculation disorders, which is consistent with the pathogenesis of “internal obstruction of blood stasis” in traditional Chinese medicine.

In this study, VAS demonstrated the strongest and most comprehensive predictive power for all aspects of SPP. It was the strongest independent risk factor for the deterioration of multiple SPP dimensions in the multivariate analysis. The patient’s pain perception is the core bridge connecting the objective disease state and their subjective health perception. Pain severely affects quality of life, daily activity ability, and social participation, and significantly increases the risk of anxiety and depression (8). The strong correlation between VAS and SDSSD is particularly notable. According to traditional Chinese medicine, “if there is no smooth flow, there will be pain.” Pain itself is an important manifestation of poor circulation of qi and blood. Persistent pain and disease activity may damage the spleen qi, leading to dysfunction in the transformation and transportation of body fluids, resulting in the syndrome of “spleen deficiency and excessive dampness.” This is manifested as fatigue, heavy limbs, and loss of appetite. Our results provide data support for this clinical observation. The RCS analysis shows that the relationship between VAS and many SPP indicators is non-linear. This means that when pain exceeds a certain critical level, its negative impact on the patient’s function may increase sharply. This emphasizes the importance of keeping pain below a certain threshold in clinical practice (33).

One of the most important findings of this study is the synergistic predictive effect of PLR and VAS on SPP. This synergistic effect was observed in multiple SPP dimensions. This suggests that inflammation and pain perception jointly exacerbate the disease burden of patients. Inflammation may directly or indirectly aggravate pain by intensifying joint damage or releasing inflammatory factors; conversely, continuous pain stress may also exacerbate neuroendocrine and immune responses, maintaining or even amplifying the inflammatory state (34). This proposed “inflammation-pain” vicious cycle is a speculative hypothesis derived from published literature and correlational data, without confirmed causal interactions. Both PLR and VAS alone have predictive value, but combining them can more accurately identify the patient group with the highest risk of SPP deterioration. The mediation analysis offers exploratory clues for understanding how PLR may be associated with SPP through the potential pathway of VAS, but these findings should be considered hypothesis-generating rather than causal proof. We found that VAS played a nearly complete mediating role between PLR and MH as well as SDSSD. This observed association only puts forward a possible correlative linkage rather than verifying a definite causal chain. This observed association only puts forward a possible correlative linkage rather than verifying a definite causal chain. This means that PLR is likely to first affect the patient’s pain experience or overall disease activity perception, and then this aggravated pain directly leads to psychological distress or the manifestation of the syndrome of “spleen deficiency and dampness excess.”

Our subgroup analysis revealed the differences in risk patterns among different patient groups. On multiple SPP indicators, the increased risk brought about by the combination of high PLR and high VAS was more significant in female patients. This might be related to the fact that women are more sensitive to pain perception, differences in hormone levels, and complex social and psychological factors (35). For patients with a disease duration of ≥10 years, under the condition of high PLR and high VAS, their risk of SAS is much higher than that of patients with a shorter disease duration. Long disease duration often implies cumulative joint damage, more complex treatment experiences and possible concurrent other health problems, all of which may lead to patients being more sensitive to pain and disease activity or a decrease in tolerance (36), Greater psychological burden (37). The BMI of the patients has a regulatory effect on the association between the high PLR + high VAS combination and certain specific SPP outcomes. The risk of SDSSD is more prominent in the group with abnormal BMI, and obesity is the main group with abnormal BMI. Adipose tissue is an active endocrine organ that secretes adipokines and pro-inflammatory factors such as leptin, resistin, IL-6, etc (38). The systemic inflammatory state reflected by high PLR is superimposed and amplified with the chronic low-grade inflammation associated with obesity. This dual inflammatory burden, combined with the disease activity represented by high VAS, is more likely to lead to metabolic disorders and gastrointestinal dysfunction, which precisely conforms to the modern pathological basis of “spleen deficiency and dysfunction in transportation, with internal generation of dampness” in traditional Chinese medicine. Moreover, obesity itself is also an important characteristic of the “phlegm-dampness constitution” in traditional Chinese medicine.

The present study has several strengths and limitations. Although classic RA activity indices such as DAS28 and CDAI, as well as routine inflammatory markers, are widely recognized gold-standard tools, direct head-to-head comparison of the PLR + VAS combination with these indices was not feasible in this retrospective study, because standardized 28-joint tender and swollen counts were incompletely recorded in electronic medical records, making reliable calculation of DAS28, CDAI and SDAI unavailable. Nevertheless, PLR + VAS possesses obvious practical advantages: PLR is derived from routine blood tests with low cost and easy accessibility, and can supplement inflammatory assessment when CRP/ESR are normal; the combination of PLR and VAS forms a concise objective inflammation + subjective symptom two-dimensional evaluation model. Moreover, PLR + VAS achieves over 90% predictive confidence for TCM syndrome-related SPP outcomes, showing better performance than single indicators in reflecting TCM characteristics. Therefore, PLR + VAS can serve as a valuable complementary stratification marker rather than a substitute for standard indices, and its incremental clinical value needs further prospective verification with complete joint count data.

The main limitations of this study are its retrospective observational design, which can only reveal statistical correlations rather than causal inference. Most notably, detailed and standardized medication information including conventional synthetic DMARDs, biological agents, targeted synthetic drugs, and glucocorticoids was not fully and consistently recorded in our retrospective real-world electronic medical database. These therapeutic interventions directly regulate systemic inflammatory status, alter peripheral blood PLR values, relieve pain and disease activity reflected by VAS, and further improve physical, psychological and TCM syndrome-related SPP outcomes. Therefore, unmeasured treatment effects may serve as important confounding factors and potentially distort the observed associations between PLR, VAS and SPP.

Two plausible confounding mechanisms exist: patients with higher baseline inflammation and pain were more likely to receive intensified treatment regimens, which may attenuate the observed associations; conversely, effective medication could simultaneously reduce PLR/VAS and ameliorate SPP, leading to residual confounding. Due to the lack of complete, unified records on medication category, dosage and treatment duration in this retrospective cohort, we were unable to conduct formal medication-based sensitivity analysis or incorporate medication-related covariates into regression models to eliminate such bias. We explicitly acknowledge this as an unavoidable major limitation of this study.

## 5 Conclusion

In conclusion, our study has confirmed the associations between PLR, VAS and SPP. High PLR and high VAS are independently associated with an increased risk of SPP deterioration, and they can jointly identify patients at high risk of SPP deterioration, with the combined identification showing stronger performance. During the process where PLR is associated with the deterioration of SPP, VAS plays a mediating role. Women, those with a long disease course (≥10 years), and those with abnormal BMI have a more significant risk of SPP deterioration. Our study has constructed a combined Chinese and Western medicine assessment framework of “inflammatory biomarker (PLR)-subjective symptoms (VAS)-multi-dimensional patient self-perception (SPP)” to support complementary risk stratification and personalized clinical evaluation of RA.

## Data Availability

The raw data supporting the conclusions of this article will be made available by the authors, without undue reservation.
