# Supplementary material for: Combined PLR and VAS for patient self-perception in rheumatoid arthritis: a retrospective study
Source: Front Med (Lausanne). 2026 Jul 1;13:1836171. doi: 10.3389/fmed.2026.1836171 (PMC13368734; doi:10.3389/fmed.2026.1836171)
Supplement: Supplementary file 1 [file Data_Sheet_1.DOCX]

Supplementary Table 1：Test Results of the Pattern of Dose-Response Relationship between PLR and VAS and Each SPP Index

| SPP Indicators | PLR relationship model (P value) | VAS Relationship Model (P value) |
| --- | --- | --- |
| PF | Linear (P-overall<0.0001, P-nonlinear>0.05) | Nonlinear (P-overall<0.001, P-nonlinear<0.05) |
| RP | - | Nonlinear (P-overall<0.001, P-nonlinear<0.05) |
| BP | Linear (P-overall<0.001, P-nonlinear>0.05) | Nonlinear (P-overall<0.001, P-nonlinear<0.05) |
| GH | Linear (P-overall<0.001, P-nonlinear>0.05) | Nonlinear (P-overall<0.001, P-nonlinear<0.05) |
| VT | Linear (P-overall<0.001, P-nonlinear>0.05) | Nonlinear (P-overall<0.001, P-nonlinear<0.05) |
| SF | Nonlinear (P-overall<0.001, P-nonlinear<0.05) | Nonlinear (P-overall<0.001, P-nonlinear<0.05) |
| RE | - | Nonlinear (P-overall<0.001, P-nonlinear<0.05) |
| MH | Linear (P-overall<0.001, P-nonlinear>0.05) | Nonlinear (P-overall<0.001, P-nonlinear<0.05) |
| SAS | Linear (P-overall<0.001, P-nonlinear>0.05) | Linear (P-overall<0.001, P-nonlinear>0.05) |
| SDS | Linear (P-overall<0.05, P-nonlinear>0.05) | Nonlinear (P-overall<0.001, P-nonlinear<0.001) |
| SDH | Linear (P-overall<0.05, P-nonlinear>0.05) | Nonlinear (P-overall<0.001, P-nonlinear<0.05) |
| SDSSD | Linear (P-overall<0.05, P-nonlinear>0.05) | Linear (P-overall<0.05, P-nonlinear>0.05) |
| SBS | Linear (P-overall<0.05, P-nonlinear>0.05) | Nonlinear (P-overall<0.001, P-nonlinear<0.001) |

Supplementary Table 2. Floor and ceiling effects of SF-36 dimensions (n=708)

| SF-36 dimension | Floor (% with score = 0) | Ceiling (% with score = 100) |
| --- | --- | --- |
| PF | 0.6% (4/708) | 0% (0/708) |
| RP | 48.2% (341/708) | 5.4% (38/708) |
| BP | 4.0% (28/708) | 14.1% (100/708) |
| GH | 1.3% (9/708) | 0.1% (1/708) |
| VT | 0% (0/708) | 0.7% (5/708) |
| SF | 3.5% (25/708) | 8.1% (57/708) |
| RE | 41.5% (294/708) | 11.6% (82/708) |
| MH | 0% (0/708) | 0.4% (3/708) |

*Note: Floor = lowest possible score (0); Ceiling = highest possible score (100).*

Supplementary Table 3. Prevalence of anxiety and depression (n=708)

| Scale | Cutoff | Abnormal count | Prevalence |
| --- | --- | --- | --- |
| SAS | ≥50 | 555 | 78.4% |
| SDS | ≥50 | 681 | 96.2% |

### Supplementary Table 4. Distribution of TCM syndrome scores (n=708)

| TCM syndrome | Median (IQR) | Min–Max |
| --- | --- | --- |
| SDH | 14 (12–16) | 4–27 |
| SDSSD | 12 (9–14) | 0–26 |
| SBS | 7 (5–8) | 0–15 |

Supplementary Table 5：The correlation between PLR, VAS and SPP risks

| Predictor variable | SPP Index | Adjusted OR (95% CI) | P value |
| --- | --- | --- | --- |
| PLR | PF | 1.67 (1.06-2.64) | 0.027 |
| VAS | PF | 5.14 (3.08-8.59) | <0.001 |
| IL-6 | PF | 1.79 (1.10-2.92) | 0.020 |
| PLR | RP | 4.54 (1.79-11.48) | 0.001 |
| Hs-CRP | RP | 3.35 (1.14-9.86) | 0.028 |
| IgG | RP | 0.39 (0.18-0.84) | 0.016 |
| VAS | BP | 3.27 (1.54-6.94) | 0.002 |
| RF | BP | 2.33 (0.95-5.72) | 0.066 |
| IgG | BP | 0.50 (0.24-1.02) | 0.055 |
| PLR | GH | 2.36 (1.09-5.12) | 0.029 |
| VAS | GH | 5.69 (2.30-14.05) | <0.001 |
| PLR | VT | 2.08 (1.19-3.63) | 0.010 |
| VAS | VT | 3.61 (1.99-6.52) | <0.001 |
| VAS | SF | 2.28 (1.63-3.21) | <0.001 |
| IL-6 | SF | 1.86 (1.27-2.73) | 0.001 |
| VAS | RE | 4.17 (2.77-6.28) | <0.001 |
| PLR | MH | 1.85 (1.25-2.74) | 0.002 |
| VAS | MH | 7.54 (4.86-11.70) | <0.001 |
| VAS | SAS | 5.95 (3.38-10.47) | <0.001 |
| VAS | SDS | 6.09 (1.30-28.47) | 0.022 |
| VAS | SDH | 3.00 (2.19-4.11) | <0.001 |
| IL-6 | SDH | 1.54 (1.06-2.22) | 0.023 |
| VAS | SDSSD | 4.17 (3.02-5.75) | <0.001 |
| PLR | SBS | 1.37 (1.00-1.88) | 0.047 |
| VAS | SBS | 2.91 (2.14-3.98) | <0.001 |

Supplementary Table 6：The combined level of PLR and VAS and its correlation with SPP risk (with the low PLR + low VAS group serving as the reference)

| SPP | Comparison group (vs. low PLR + low VAS) | Adjust OR (95% CI) | P value |
| --- | --- | --- | --- |
| PF | HighPLR + HighVAS | 7.74 (3.78-15.85) | <0.001 |
|  | HighPLR + LowVAS | 1.83 (1.07-3.11) | 0.026 |
|  | LowPLR + HighVAS | 5.96 (2.97-11.94) | <0.001 |
| RP | HighPLR + HighVAS | 3.31 (1.27-8.63) | <0.001 |
| BP | HighPLR + HighVAS | 4.02 (1.29-12.52) | 0.017 |
| GH | HighPLR + HighVAS | 5.66 (2.24-14.30) | <0.001 |
|  | HighPLR + LowVAS | 3.74 (1.48-9.48) | 0.005 |
|  | LowPLR + HighVAS | 4.20 (1.66-10.61) | 0.002 |
| VT | HighPLR + HighVAS | 6.73 (2.72-16.66) | <0.001 |
|  | LowPLR + HighVAS | 2.40 (1.21-4.77) | 0.012 |
| SF | HighPLR + HighVAS | 2.55 (1.61-4.03) | <0.001 |
|  | LowPLR + HighVAS | 2.11 (1.32-3.37) | 0.002 |
| RE | HighPLR + HighVAS | 4.74 (2.69-8.36) | <0.001 |
|  | LowPLR + HighVAS | 3.72 (2.11-6.57) | <0.001 |
| MH | HighPLR + HighVAS | 9.99 (5.55-17.98) | <0.001 |
|  | HighPLR + LowVAS | 1.74 (1.10-2.75) | 0.018 |
|  | LowPLR + HighVAS | 6.91 (3.92-12.18) | <0.001 |
| SAS | HighPLR + HighVAS | 7.15 (3.43-14.91) | <0.001 |
|  | LowPLR + HighVAS | 5.84 (2.72-12.53) | <0.001 |
| SDS | HighPLR + HighVAS | 6.67 (1.90-23.48) | 0.003 |
|  | LowPLR + HighVAS | 3.16 (1.12-8.85) | 0.029 |
| SDH | HighPLR + HighVAS | 3.26 (2.13-4.98) | <0.001 |
|  | LowPLR + HighVAS | 2.93 (1.89-4.56) | <0.001 |
| SDSSD | HighPLR + HighVAS | 7.45 (4.70-11.81) | <0.001 |
|  | HighPLR + LowVAS | 1.85 (1.18-2.88) | 0.007 |
|  | LowPLR + HighVAS | 3.48 (2.23-5.41) | <0.001 |
| SBS | HighPLR + HighVAS | 4.01 (2.62-6.14) | <0.001 |
|  | HighPLR + LowVAS | 1.55 (0.99-2.42) | 0.055 |
|  | LowPLR + HighVAS | 3.27 (2.11-5.08) | <0.001 |

Supplementary Table 7：Association Rules Analysis of PLR、VAS and Various SPP Indicators

| Antecedent | Consequent | Support (%) | Confidence (%) | Lift |
| --- | --- | --- | --- | --- |
| PLR | PF | 49.86 | 89.52 | 1.02 |
|  | RP | 49.86 | 98.30 | 1.01 |
|  | BP | 49.86 | 95.18 | 1.01 |
|  | GH | 49.86 | 99.15 | 1.01 |
|  | VT | 49.86 | 94.05 | 1.02 |
|  | SF | 49.86 | 72.52 | 1.04 |
|  | RE | 49.86 | 81.02 | 1.03 |
|  | MH | 49.86 | 82.44 | 1.08 |
|  | SAS | 49.86 | 89.80 | 1.03 |
|  | SDS | 49.86 | 98.58 | 1.01 |
|  | SDH | 49.86 | 60.06 | 1.06 |
|  | SDSSD | 49.86 | 57.79 | 1.08 |
|  | SBS | 49.86 | 56.94 | 1.09 |
| Antecedent | Consequent | Support (%) | Confidence (%) | Lift |
| VAS | PF | 52.26 | 94.32 | 1.07 |
|  | RP | 52.26 | 99.73 | 1.02 |
|  | BP | 52.26 | 97.30 | 1.03 |
|  | GH | 52.26 | 99.73 | 1.01 |
|  | VT | 52.26 | 98.38 | 1.07 |
|  | SF | 52.26 | 78.38 | 1.12 |
|  | RE | 52.26 | 89.73 | 1.14 |
|  | MH | 52.26 | 91.62 | 1.20 |
|  | SAS | 52.26 | 95.41 | 1.09 |
|  | SDS | 52.26 | 99.46 | 1.01 |
|  | SDH | 52.26 | 69.73 | 1.23 |
|  | SDSSD | 52.26 | 70.00 | 1.21 |
|  | SBS | 52.26 | 64.86 | 1.18 |

Supplementary Table 8：Association rule analysis of the correlation between PLR combined with VAS and various SPP indicators

| **Antecedent** | **Consequent** | **Support (%)** | **Confidence (%)** | **Lift** |
| --- | --- | --- | --- | --- |
| PLR &VAS | PF | 50.27 | 61.62 | 1.15 |
|  | RP | 50.27 | 89.18 | 1.06 |
|  | BP | 50.27 | 79.45 | 1.07 |
|  | GH | 50.27 | 85.40 | 1.09 |
|  | VT | 50.27 | 62.16 | 1.11 |
|  | SF | 50.27 | 90.27 | 1.12 |
|  | RE | 50.27 | 88.10 | 1.16 |
|  | MH | 50.27 | 90.27 | 1.12 |
|  | SAS | 50.27 | 94.59 | 1.07 |
|  | SDS | 50.27 | 94.59 | 1.08 |
|  | SDH | 50.27 | 91.35 | 1.06 |
|  | SDSSD | 50.27 | 91.35 | 1.26 |
|  | SBS | 50.27 | 90.81 | 1.18 |

Supplementary Table 9: Summary Table of the Potential Mediating Role of VAS

| X~M~Y | c | a | b | a*b | a*b | a*b | a*b | c' | Inspection conclusion |
| --- | --- | --- | --- | --- | --- | --- | --- | --- | --- |
|  | Total effect |  |  | Interme diary effect value | (Boot SE) | (p value) | (95% BootCI) | Direct effect |  |
| PLR~VAS~PF | -0.51 | 0.63 | -0.52 | -0.328 | 0.06 | <0.001 | [-0.45, -0.22] | -0.14* | Partial mediation |
| PLR~VAS~RP | -0.64 | 0.65 | -0.71 | -0.462 | 0.08 | <0.001 | [-0.62, -0.32] | -0.16* | NO mediation |
| PLR~VAS~BP | -0.36 | 0.59 | -0.46 | -0.271 | 0.05 | <0.001 | [-0.37, -0.18] | -0.11 | Partial mediation |
| PLR~VAS~GH | -0.48 | 0.61 | -0.50 | -0.305 | 0.05 | <0.001 | [-0.41, -0.21] | -0.14* | Partial mediation |
| PLR~VAS~VT | -0.35 | 0.58 | -0.44 | -0.255 | 0.04 | <0.001 | [-0.34, -0.18] | -0.11 | Partial mediation |
| PLR~VAS~SF | -0.44 | 0.60 | -0.49 | -0.294 | 0.05 | <0.001 | [-0.39, -0.20] | -0.13* | Partial mediation |
| PLR~VAS~RE | -0.45 | 0.66 | -0.57 | -0.376 | 0.07 | <0.001 | [-0.51, -0.25] | -0.12 | NO mediation |
| PLR~VAS~MH | -0.50 | 0.65 | -0.69 | -0.449 | 0.04 | <0.001 | [-0.53, -0.37] | -0.01 | Complete mediation |
| PLR~VAS~SAS | -0.56 | 0.68 | -0.67 | -0.456 | 0.08 | <0.001 | [-0.61, -0.32] | -0.12* | Partial mediation |
| PLR~VAS~SDS | -0.55 | 0.64 | -0.55 | -0.352 | 0.06 | <0.001 | [-0.46, -0.24] | -0.16* | Partial mediation |
| PLR~VAS~SDH | -0.48 | 0.60 | -0.45 | -0.270 | 0.03 | <0.001 | [-0.33，-0.21] | -0.18* | Partial mediation |
| PLR~VAS~SDSSD | -0.47 | 0.62 | -0.53 | -0.329 | 0.06 | <0.001 | [-0.45, -0.22] | -0.15* | Complete mediation |
| PLR~VAS~SBS | -0.43 | 0.59 | -0.48 | -0.283 | 0.05 | <0.001 | [-0.38, -0.19] | -0.12 | Partial mediation |

Supplementary figure1: Correlation analysis chord diagram of PLR, VAS and SPP indicators


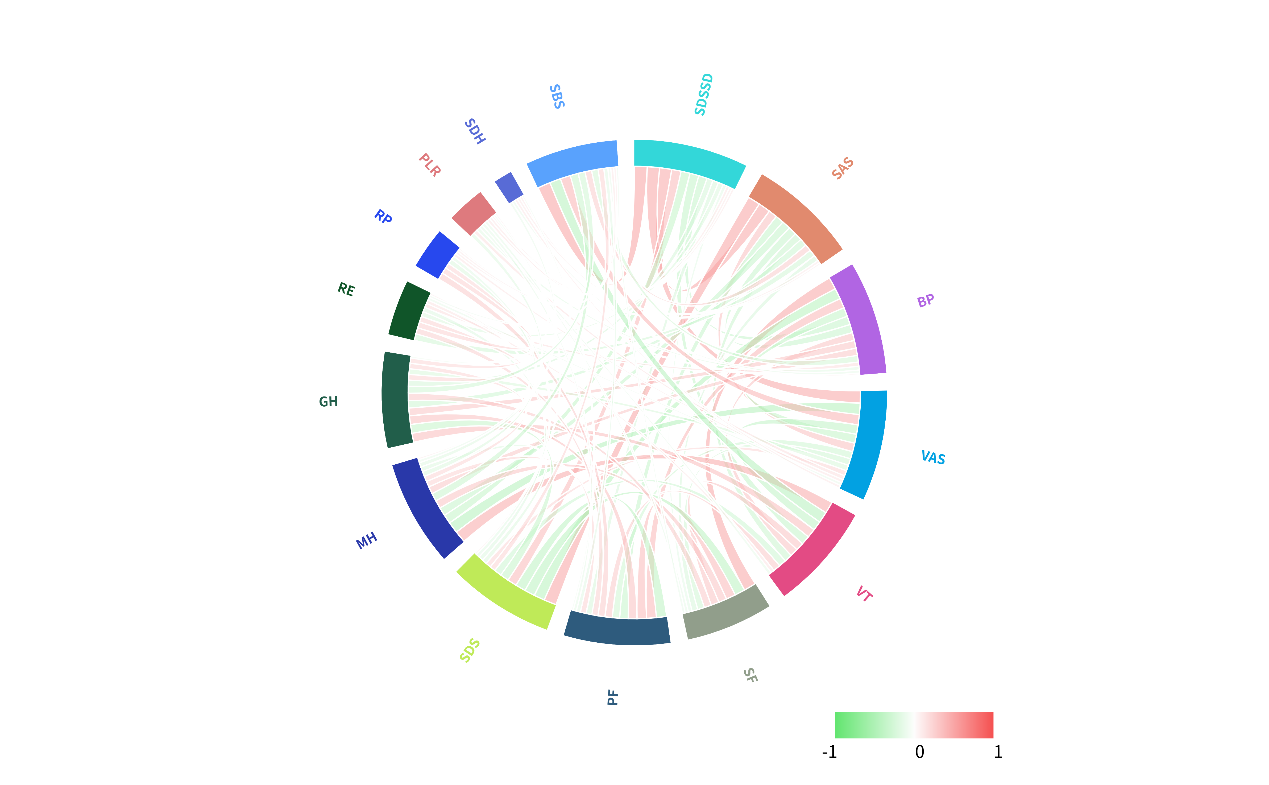


Supplementary figure 2:Association rule analysis diagram.A: Visualized Graph of Association Rules between PLR, VAS and Each SPP Indicator;B:Visualization graph of association rules between PLR combined with VAS and various SPP indicators


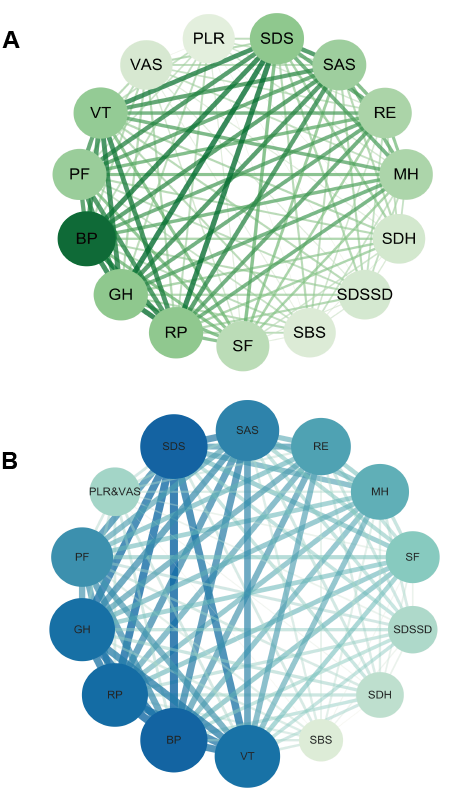


Supplementary figure 3: Heatmap showing the correlations between gender, age, BMI, disease duration and PLR, VAS and SPP indicators


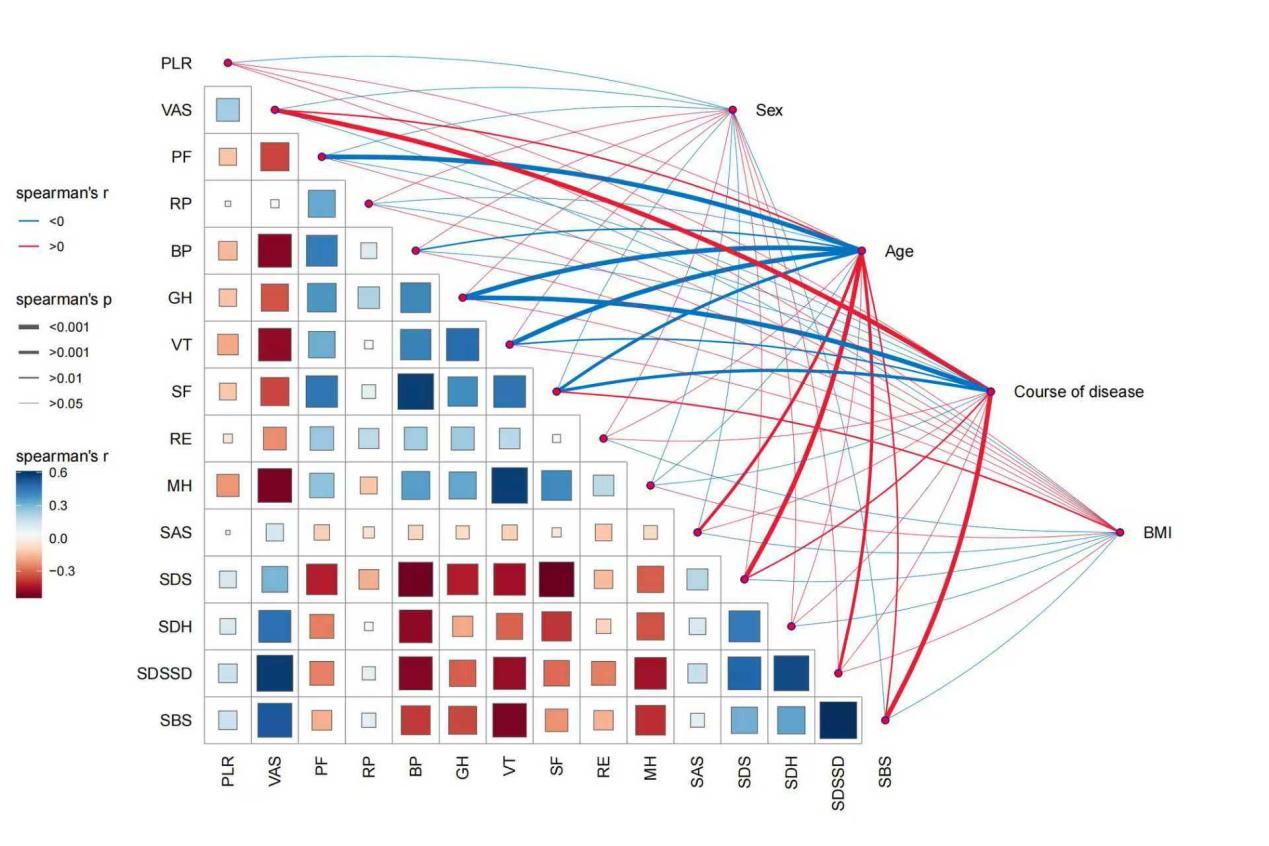


:
